# 189. Bacteremia among COVID-19 and Non-Covid-19 Patients Admitted in the ICU

**DOI:** 10.1093/ofid/ofab466.391

**Published:** 2021-12-04

**Authors:** paraskeui chra, Evdokia Gavrielatou, prodromos Temperikidis, Michalis Tsimaras, eleni magira

**Affiliations:** 1 Evanelismos Hospital, Athens, Attiki, Greece; 2 National Kapodistrian University of Athens, Athens, Attiki, Greece; 3 University of Athens Medical School, athens 15235, Attiki, Greece

## Abstract

**Background:**

The aim of this work were to investigate the rate and aetiology of bloodstream infection collected from COVID and non-COVID patients admitted in the ICU

**Methods:**

A retrospective cohort study was conducted on PCR Covid-19 positive patients admitted in the ICU from 20th March to 30^th^ April 2020. Corresponding data from the same period in 2019 collected of all consecutive patients admitted in the same ICU were retrospectively reviewed for the presence of microbiologically documented bloodstream infections at least 8 hours after admission. All patients in the cohort study were on mechanical ventilation, or at some point during their ICU admission required mechanical ventilation.

**Results:**

We identified a total of 19 (38%) BSIs in the COVID-19 group and 10 (12%) BSI in the non-COVID-19 group (*p*=0,8). COVID-19 patients had an increased probability to develop ICU-BSI, at a median of 8 days of ICU admission as opposed to 6 in the non-COVID-19 group. Patients were comparable in terms of age, and APACHE II score. Out of 19 BSI CoVID-19 patients, 14 (73%) were male vs 5 (50%) in the non-CoVID-19 BSI patients (*p*=0.0007). Of all BSI-CoVID-19 patients, 7 cases (37%), 3 (16%), and 3(16%) had underlying diseases such as hypertension, diabetes, and obesity vs 1(9%), 0(0%), and 0 (0%) in the BSI-non CoVID-19 patients statistically significant at p=0.004, p=0.05, and p=0.05, respectively. ICU-acquired BSIs were mostly due to multi-drug-resistant pathogens. Clinical outcomes were statistically significantly different between patients with CoVid-19 BSI 7(37% ) and 2(20%)in BSI- non-CoVID-19 pneumonia (p=0.02).

**Conclusion:**

Our findings emphasize that although the incidence of BSI in CoVID-19 positive ICU admitted patients slightly increased their impact on overall outcome was significantly worse. Consequently, it is important to pay attention to bacterial superinfections in critical patients positive for COVID-19.

**Disclosures:**

**All Authors**: No reported disclosures

